# Disease Recognition in X-ray Images with Doctor Consultation-Inspired Model

**DOI:** 10.3390/jimaging8120323

**Published:** 2022-12-05

**Authors:** Kim Anh Phung, Thuan Trong Nguyen, Nileshkumar Wangad, Samah Baraheem, Nguyen D. Vo, Khang Nguyen

**Affiliations:** 1 Department of Computer Science, University of Dayton, Dayton, OH 45469, USA; 2 Faculty of Software Engineering, University of Information Technology, Linh Trung Ward, Thu Duc District, Ho Chi Minh City 70000, Vietnam

**Keywords:** disease recognition, medical image processing, doctor consultation-inspired

## Abstract

The application of chest X-ray imaging for early disease screening is attracting interest from the computer vision and deep learning community. To date, various deep learning models have been applied in X-ray image analysis. However, models perform inconsistently depending on the dataset. In this paper, we consider each individual model as a medical doctor. We then propose a doctor consultation-inspired method that fuses multiple models. In particular, we consider both early and late fusion mechanisms for consultation. The early fusion mechanism combines the deep learned features from multiple models, whereas the late fusion method combines the confidence scores of all individual models. Experiments on two X-ray imaging datasets demonstrate the superiority of the proposed method relative to baseline. The experimental results also show that early consultation consistently outperforms the late consultation mechanism in both benchmark datasets. In particular, the early doctor consultation-inspired model outperforms all individual models by a large margin, i.e., 3.03 and 1.86 in terms of accuracy in the UIT COVID-19 and chest X-ray datasets, respectively.

## 1. Introduction

Coronavirus disease 2019 (COVID-19) [1] is a contagious disease caused by a coronavirus called SARS CoV-2. This disease quickly spread worldwide, causing a global pandemic. Symptoms of COVID-19 appear 2–4 days after exposure. People with COVID-19 may experience fever or chills, cough or shortness of breath, breathing difficulties, headache, fatigue, and loss of smell or taste. According to the World Health Organization, COVID-19 infection can be detected by testing specimens from nose or mouth swabs. Real-time reverse transcription-polymerase chain reaction (RT-PCR) is used to detect nucleic acids in secretory fluids obtained from specimens. Because coinfection with other viruses can impact RT-PCR prediction performance, repetitive testing may be recommended to prevent false negatives. The RT–PCR test has a three-day turnaround time, as RT–PCR test tools have been scarce in recent months. There has been a pressing need for additional procedures to quickly and reliably identify COVID-19 patients. Furthermore, the swabbing operation is highly susceptible to expert errors, and it must be performed repeatedly [2]. Therefore, X-rays or CT scans of the chests are suitable complements to RT-PCR because they can be gathered and processed considerably more quickly [3]. Relative to CT scans, taking chest X-ray images is less expensive, radiation-exposing, and time-consuming. In addition, CT nuclear scanning delivers larger radiation doses than traditional X-rays scanning [4]. An X-ray of the chest produces 0.1 mSv, whereas a CT produces 70 times the amount. X-ray machines are widely available and quickly provide images for diagnosis. Thus, in this work, we focus on recognizing diseases in X-ray images.

Since the reintroduction of convolution neural networks (CNN) [5], deep learning has become dominant in many research fields, such as computer vision, natural language processing, and video/speech recognition. To date, deep learning has been adopted by a wide range of applications, owing to its scalability, speed, and efficiency, even outperforming humans in specific industrial processes [6]. As reviewed in [7], medical science is a relatively new field that is attempting to leverage the success of artificial intelligence and deep learning models. Developments in digital data collection, computer vision, and computation infrastructure have enabled AI applications to move into areas that were previously regarded as entirely human domains [8]. Deep learning in radiology is a game changer in terms of both quality and quantity when it comes to biomedical imaging explanation and data processing. Although machine learning and deep learning algorithms have demonstrated their ability to classify tumors and cancer progression, radiologists are still hesitant to use them [9]. One of the numerous advantages of machine learning in radiology is its capacity to automate or even replace radiologist scanning methods. Deep learning algorithms produce outcomes that are comparable to those of a top radiologist. However, situations in which resources are limited and requirements are particularly demanding, such as the COVID-19 pandemic, exemplify the need for a robust algorithm to assist medical professionals. 

Having witnessed the extraordinary performance of deep learning in various tasks [10,11,12,13,14,15,16,17,18,19,20,21,22], we investigated deep learning models in this paper. Inspired by the efforts and experience of healthcare professionals such as doctors and specialists during the pandemic, we propose a doctor consultation-inspired model to fuse various deep learning models to produce accurate outputs. 

The novelty of this work is as follows. First, the proposed framework is motivated from the perspective of physicians. The doctor consultation-inspired method is formulated in the form of fusion models. The proposed method considers each individual deep learning model as a medical doctor. Then, a consultation is performed based on inputs from multiple individual models. In this regard, the proposed method leverages the strengths of available methods in order to boost the performance. Second, the proposed method is open in the sense that any future individual methods can be integrated into our method. Third, we evaluate the proposed method on two benchmark datasets with different consultation modes, namely early consultation and late consultation. 

The remainder of this paper is organized as follows. In Section 2, we summarize related works. In Section 3, we introduces the proposed doctor consultation-inspired model. The experiments and the experimental results are presented and discussed in Section 4. Finally, Section 5 concludes the paper. 

## 2. Related Works

Many efforts to diagnose COVID-19 and pneumonia from X-ray images have been reported in the literature. In [10], deep neural network techniques were used in conjunction with X-ray imaging to identify COVID-19 infection. The main goal of this effort was to help alleviate doctor shortages in rural areas by providing resources to fill the gap. Shibly et al. [10] used VGG-16 [11] architecture to identify COVID-19 patients from chest X-ray images. The proposed method may aid medical professionals in screening COVID-19 patients. In another work, Sethy et al. [12] sought to detect coronavirus-infected patients using X-ray images. This method involves radiographic analysis using support vector machines (SVMs) with deep features extracted from ResNet50 [13]. The efficacy of a multi-CNN in automatically detecting COVID-19 from X-ray images was examined by Abraham et al. [14], who employed naive Bayes, SVM, AdaBoost, logistic regression, and random forests before settling on the Bayes net. The best-performing method is Xception [15]. Mei et al. [16] proposed a machine learning strategy that uses diagnostic imaging and clinical studies to accurately detect COVID-19-positive patients. The authors created a DCNN to learn the initial imaging characteristics of COVID-19 patients (18-layer residual network: ResNet-18 [13]). In the next stage, random forest, SVM, and MLP classifiers were used to categorize COVID-19 patients. Multilayer perceptron (MLP) performed best on the tuning set, and a neural network model was utilized to evaluate COVID-19 status based on radiographic and clinical data. 

Hurt et al. [17] improved their method by just using frontal chest X-ray images. They discovered that the probabilities in their model that are matched to the quality of the imaging data are remarkably general and reliable. According to recent research, machine learning algorithms can distinguish COVID-19 from other pneumonia strains. Tuncer et al. [18] developed a technique for COVID-19 recognition using X-ray scans of the lungs. This technique is broken down into stages, which are detailed as follows. Residual example local binary pattern [19] is the name given to this method (ResExLBP). In the feature selection step, the IRF-based attribute selection method is used. Decision trees, linear classifiers, SVM, k-NN, and SD approaches are utilized in the classification step. Using 10-fold cross validation, the SVM classifier once again achieved the best performance. 

Recently, Hemdan et al. [20] developed a deep learning framework to aid radiologists in detecting COVID-19 in X-ray scan. They investigated many deep artificial neural networks to classify the patient’s COVID-19 status as negative or positive. Machine learning classifiers VGG19 [11] and DenseNet201 [21] achieved the best results in predicting COVID-19 using two-dimensional X-ray images. Recently, a rapid COVID-19 diagnosis technique was proposed by Ardakani et al. [22]. The authors used ten well-known pre-trained CNNs for this purpose. They trained and tested the 10 CNNs using the same dataset and compared the results to a radiologist’s classifications. For COVID-19 individuals, ResNet-101 [13] achieved the best performance. Additionally, there are many deep learning models proposed for classification [23,24].

Many optimization and refinement steps have been proposed to improve the performance of classifiers. For example, data augmentation [5] enhances the size and quality of training datasets. Waheed et al. [25] proposed a GAN-based model to synthesize medical images, with the aim of increasing the number of training samples required to train a CNN-based model to detect COVID-19 from medical images. In another study, Oh et al. [26] proposed a patch-based deep neural network architecture that can be trained with a small dataset. Teixeira et al. [27] used a UNet-based lung segmentation model [28] to segment the lung first. Then, they used a CNN-based model to classify X-ray images. Similarly, Tartaglione et al. [29] adopted segmented lung images. Then, they used a feature extractor pretrained on CXR pathology datasets and fine-tuned it on COVID datasets. Balaha et al. [30] introduced a framework with a segmentation phase to segment lung regions. Then, data augmentation such as rotation, skewing, translation, and shifting was applied. Finally, a genetic algorithm was used to learn combinations of hyperparameters. Baghdadi et al. [31] presented an algorithm for COVID-19 classification using a CNN, pre-trained model, and Sparrow search algorithm on CT lung images. Perumal et al. [32] proposed a transfer learning model with Haralick features [33] to speed up the prediction process and assist medical professionals. Transfer learning alleviated the problem of the lack of COVID-19-positive data to some extent. A comparison of related works provided in Table 1. However, a review of all models used for COVID-19 detection is beyond the scope of this paper. Additional research works involving COVID-19, CNNs, and data augmentation were covered in [34,35,36].

## 3. Proposed Framework

### 3.1. Individual Doctor Models

**Figure 1:**
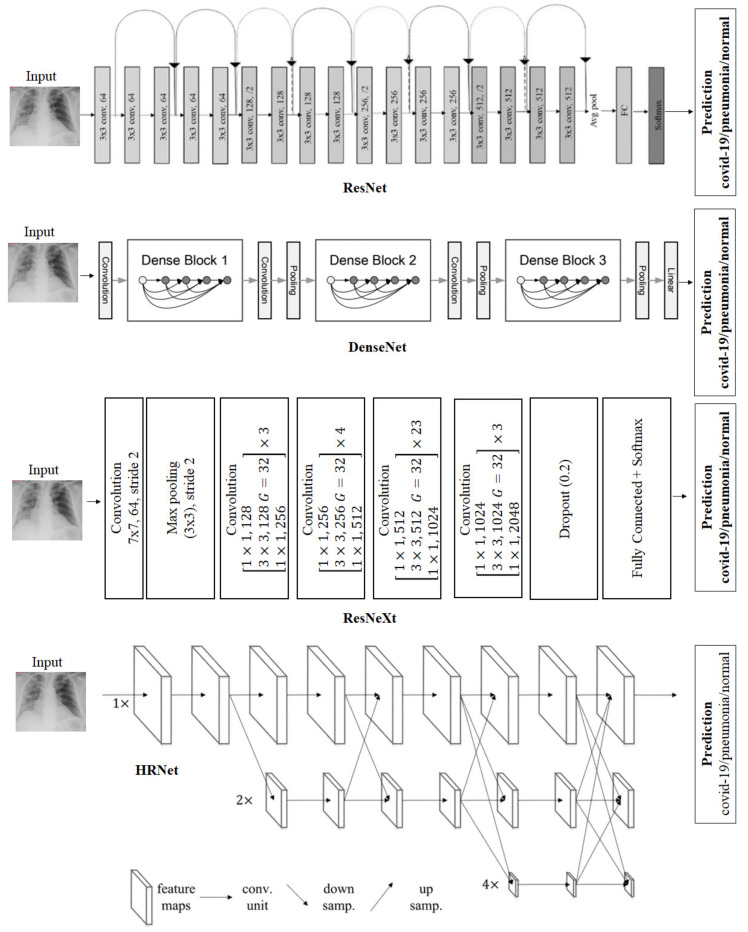
Architecture of deep learning models referred to as individual doctors utilized in our framework. From top to bottom: ResNet, DenseNet, ResNeXt, and HRNet.

In this work, we use the aforementioned deep learning models to simulate medical doctors. Figure 1 shows the architecture of the deep learning models. We adopt the available source code of these models for implementation. 

He et al. [13] found that it is very difficult to train deep neural networks, indicating that models struggle with saturation and are very difficult to optimize. Therefore, they proposed a framework to reduce training via residual learning called ResNet. In particular, ResNet considers previous layers as the input layer reformulated as learning residual functions. Many ResNet variants have been developed, with the main difference being the number of layers. for example, ResNet-18, -50, -101, and -152. 

Huang et al. [2] proposed a dense convolutional network (DenseNet), which connects each layer to every other layer in a feed-forward fashion. In particular, DenseNet distills the insight of a simple connectivity pattern, which optimizes information flow between layers and then directly connects all layers. DenseNet directly connects any layer to all subsequent layers to further improve the information flow between layers. In particular, the l-th layer has l inputs, consisting of the feature maps of all previous convolutional blocks. Then, the feature maps are passed on to all L-l subsequent layers, introducing L(L+1)/2 connections in an L-layer network. We investigate two DenseNet variants, namely DenseNet-169 and DenseNet-201.

Xie et al. [23] presented a strategy to expose a dimension called “cardinality” (i.e., the size of the set of transformations). They proposed ResNeXt, including a stack of residual blocks. ResNeXt is homogeneous and multibranched, with only a few hyperparameters to set. ResNeXt’s blocks follow two simple rules: the blocks share hyperparameters (width and filter sizes), and the width is multiplied by a factor of two. These rules are constructed while producing spatial maps of the same size and downsampling the spatial map by a factor of two each time. In addition, ResNeXt introduces the revisiting of simple neurons, which is the elementary transformation performed by fully-connected and convolutional layers. In particular, the inner product is a form of aggregating transformation. The analysis of a simple neuron replaces the elementary transformation with a more generic function. In this paper, we consider the widely used ResNeXt-101 variant.

Most state-of-the-art image classification [13,21,23] models follow the same general framework, which first encodes the image to achieve a low-resolution representation and then recovers the high-resolution representation. Wang et al. [24] proposed a framework with a high-resolution network (HRNet). HRNet maintains high-resolution representations throughout the whole process. In particular, it contains parallel multiresolution convolutions and multiresolution fusions. Parallel multiresolution convolutions start from a high-resolution convolution stream and gradually add high-to-low-resolution streams one by one, constructing new stages. Finally, it connects the multiresolution streams in parallel. As a result, the resolutions of a later stage consist of the resolutions from the previous stages. HRNet introduces repeated multiresolution fusions that exchange information across multiresolution representations. In our experiment, we leverage HRNetV2-W48, with a high-resolution width of 48. 

### 3.2. Doctor Consultation-Inspired Model

In this work, multiple deep learning models are used to recognize diseases such as pneumonia and COVID-19 in X-ray images. In reality, a consultation session allows a team of healthcare professionals such as doctors and specialists to limit the damage of the acute respiratory distress syndrome (ARDS), evaluate recovery, manage lingering symptoms, and prevent a recurrence in the future. Follow-up is recommended post COVID syndrome to help the patient get back on track. Inspired by such consultation sessions, in this work, we consider each model as a doctor. Then, we combine the decisions of the various models to output diagnostic and prognostic results. There are two strategies available for doctor consultation models, namely late consultation and early consultation. The details are provided below.

**Late Consultation.** In the late consultation model, each doctor makes his/her own final decision. The consultation simply combines all of these decisions. In our method, to simulate this strategy, we fuse the prediction scores from individual models to output a final decision. In particular, we first train n  individual models on the training data. Then, we feed the images in the training set to each model (i) to output a prediction score ($p_{i}$), where $p_{i}$ is a vector containing m values corresponding to m disease classes. We consider $p_{i}$ as the *i*th doctor’s final decision. We further train a classification model ($f_{late}$) to output the final decision (${\hat{y}}_{late}$), as shown in Equation (1):

(1) $${\hat{y}}_{late}=f_{late}(\left[p_{1}\left|\left|p_{2}\right|\right|\ldots \left|\right|p_{n}]\right).$$

In Equation (1), [.||.] denotes the concatenate operation. 

**Early Consultation.** For the early consultation strategy, the decision is made based on the observations and discussions among all health professionals in the consultation. To simulate this strategy, we first train n individual models on the training data. Then, we feed the images in the training set to each model (i). Instead of obtaining the prediction scores ($p_{i}s$), we fetch the deep-learned features ($x_{i}$) of the individual model (i). The deep-learned features are extracted in the layer prior to the fully connected layer. The deep-learned features have been shown to be effective in classification tasks [5,37,38]. We normalize each individual deep-learned feature with $l_{2}$ normalization. We consider $x_{i}$ as the *i*th doctor’s observation/discussion. We further train a classification model ($f_{early}$) to output the final decision (${\hat{y}}_{early}$), as expressed by Equation (2):

(2) $${\hat{y}}_{early}=f_{early}([x_{1}||\;x_{2}\left|\left|\;\ldots \right|\right|x_{n}]).$$

For the classification model such, i.e., $f_{late}$ or $f_{early}$, we adopt support vector machine (SVM), which is popular for COVID-19 recognition tasks [12,15,16,18]. Specifically, SVM seeks a hyperplane in a high-dimensional space to maximize the margin between classes. 

**Figure 2:**
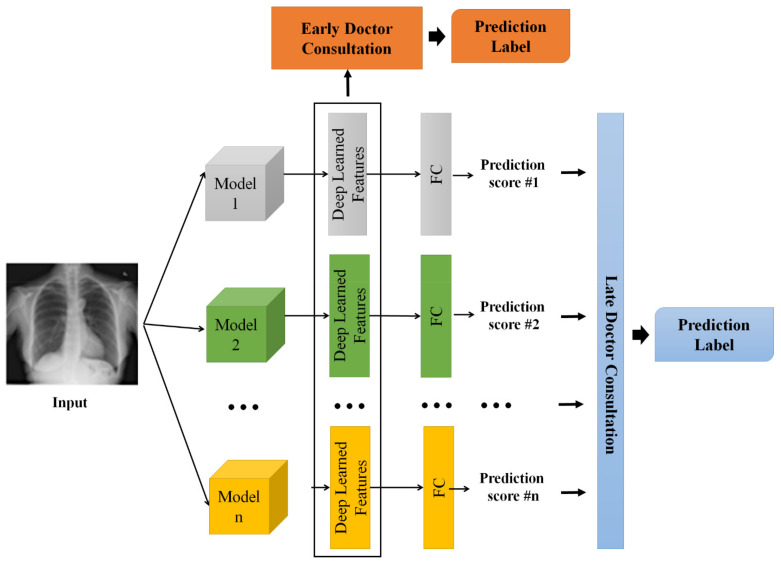
The framework of the proposed doctor consultation-inspired model. In particular, there are two fusion mechanisms, namely early fusion and late fusion. The final output of either mechanism is the prediction label.

Figure 2 illustrates the two aforementioned consultation strategies. In this work, we investigate both consultation strategies in the evaluation. For reading clarity, the abbreviations and symbols used are listed on Table 2 and Table 3, respectively.

## 4. Experiments

### 4.1. Experimental Settings

In this work, we first use the available UIT COVID-19 dataset [39]. This dataset consists of 1317 images annotated in 3 classes, namely COVID-19, pneumonia, and normal. Because COVID-19 illness symptoms are similar to pneumonia symptoms, and pneumonia disease is responsible for many of COVID-19 virus-related deaths, it is logical to combine the two diagnostic techniques. The two subsets, i.e., the training set and testing set, comprise 1053 and 264 images, respectively. 

We also conduct experiments on a chest X-ray dataset [40]. This dataset consists of 6432 X-ray images with 3 classes: COVID-19, pneumonia, and normal. The dataset is organized into 2 subsets, namely a training set and a testing set. In particular, there are 5144 images in the training set and 1288 images in the testing set.

Regarding the performance metrics, we report the results in terms of accuracy, precision, recall, and *F*1 score. In particular, Accuracy describes the number of correct predictions over all predictions.

(3) $$Accuracy=\frac{True\;Positive+True\;Negative\;}{True\;Positive+False\;Positive+True\;Negative+False\;Negative}.$$

Here, a true/false positive is an outcome for which the model correctly/incorrectly predicts the positive class. Similarly, a true/false negative is an outcome for which the model correctly/incorrectly predicts the negative class. The second metric, Precision, is a measure of true positives.

(4) $$Precision=\frac{True\;Positive}{True\;Positive+False\;Positive}.$$

Recall is a measure of the number of correctly predicted positive cases over all positive cases in the dataset.

(5) $$Recall=\frac{True\;Positive}{True\;Positive+False\;Negative}.$$

*F*1 score is a measure combining both precision and recall. It is generally described as the harmonic mean of the two. The formula for the *F*1 score is expressed as:

(6) $$F1=2\times \frac{Precision\times Recall\;}{Precision+Recall}.$$

We conduct our experiments on a CPU Intel (R) Core(TM) i9-10900X CPU @ 3 with 64 GB of RAM and one GeForce RTX 2080 Ti 12GB. The experimental configurations are primarily leveraged from MMClassification Toolbox and Benchmark version 0.24.0 based on PyTorch V1.8.1 version [41]. In particular, we adopt the configuration achieving the best performance on the Image-Net classification task. The SVM classifier is trained by using the scikit-learn library.

### 4.2. Experimental Results

We first conduct an ablation study to evaluate the performance of individual doctor models and the proposed doctor consultation-inspired method with both early and late consultation mechanisms. 

Table 4 shows the performance of the various methods on the UIT COVID-19 benchmark dataset [39] in terms of accuracy, precision, recall, and *F*1 score, as described in Equations (3)–(6), respectively. Generally, complicated models achieve superior performance. For example, ResNet-152 performs better than other ResNet variants. Among the individual models, DenseNet-201 and HRNet obtain the top performance, owing to their advanced architectures. The doctor consultation models achieve better performance than the individual models, indicating the effectiveness of the proposed method for the task of anomaly analysis in medical image processing. The early consultation model outperforms the late consultation model, i.e., 94.32, 94.36, 94.32, and 94.31 vs. 92.42, 92.42, 92.42, and 92.42 in terms of accuracy, precision, recall, and *F*1 score, respectively. These results imply that the concatenation of features from individual doctor models is useful in making a final prediction. The fusional nature of the late consultation model may be biased by the “good” individual models, for example, HRNet or DenseNet.

We then conduct experiments on the chest X-ray dataset [40]. The results are shown in Table 5. DenseNet-201 and HRNet-W48 achieve the top-2 performance among the individual models, i.e., 93.17 and 92.62 in terms of accuracy, respectively. Unlike the UIT COVID-19 dataset, the ResNet variants are outperformed by ResNeXt-101 on this benchmark. The late consultation and early consultation mechanisms obtain the top-2 highest scores across all metrics. The early consultation mode once again surpasses the late consultation model. 

**Figure 3:**
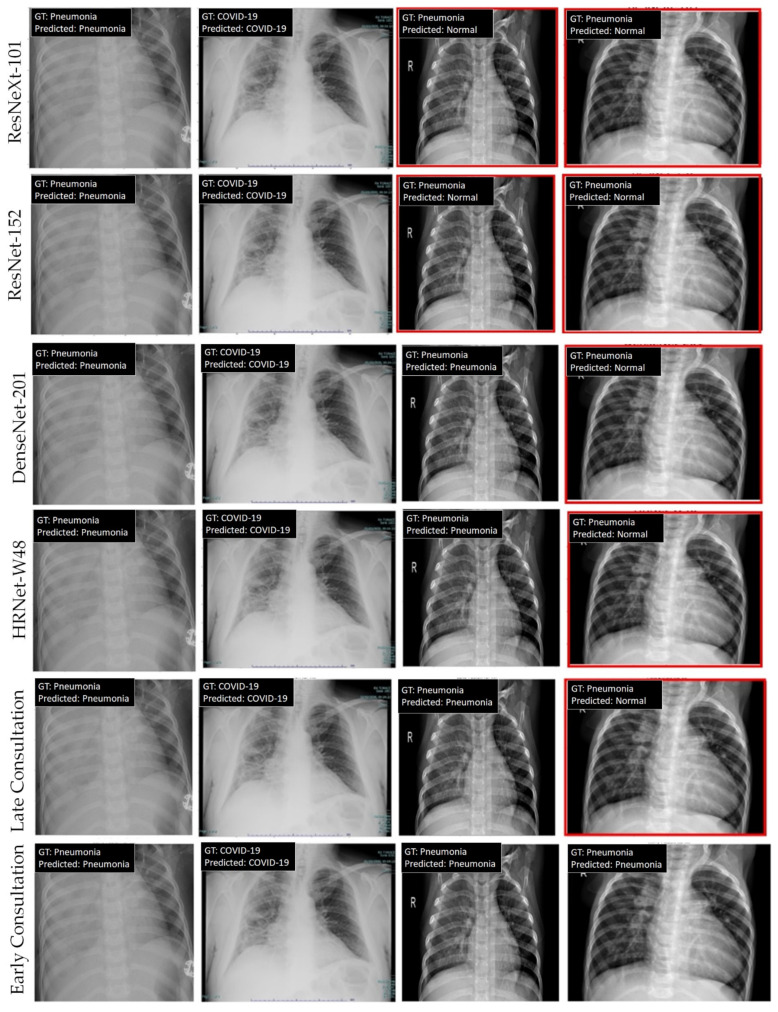
Visualization of the prediction results of various models (best viewed online in color with zoom). From top to bottom: ResNeXt101, ResNet152, DenseNet201, HRNet-W48, late consultation, and early consultation. Incorrect predictions are marked with a red rectangle.

We further visualize the classification results of the various methods in the UIT COVID-19 benchmark. Figure 3 shows the visualization of the prediction results from the baselines and our two consultation modes. As shown in the figure, all models perform well in predicting the results in the first two columns. The third column shows failure predictions of individual models. However, both consultation models output correct predictions. The last column demonstrates the advantage of the early consultation over the late consultation strategy. In particular, whereas the late consultation follows all incorrect decisions of individual models, the early consultation model yields the correct prediction, indicating the effectiveness of the proposed model in handling difficult cases. 

We observe the consistent performance of the early consultation mode in both UIT COVID-19 and Chest X-ray datasets, outperforming all individual models by a large margin, i.e., 3.03 and 1.86 in terms of accuracy on the UIT COVID-19 and chest X-ray datasets, respectively. However, the individual models are inconsistent. For example, ResNet-50 does not perform well on the UIT COVID-19 dataset but achieves a high performance on the chest X-ray dataset. Furthermore, we evaluate the performance of state-of-the-art baselines on the two benchmark datasets. As shown in Table 6, our proposed method achieves the best performance on both sets. Specifically, the early consultation method outperforms the late consultation method. The baselines are inconsistent between both sets. Here, we would like to highlight the limitations of the proposed work. First, the performance of the late and early consultation models heavily relies on the performance of the individual models. If all of individual models achieve a low performance, this hurts the overall performance of the doctor consultation-inspired model. Second, because SVM is adopted for fusion, the proposed framework lacks explainability. 

## 5. Conclusions

In this paper, we propose a doctor consultation-inspired method for recognizing disease from X-ray images. Inspired by doctor consultation practice, we explore two modes, namely late fusion and early fusion. The proposed method takes advantage of multiple state-of-the-art networks to efficiently recognize disease from an input X-ray image. The early fusion mechanism combines the deep-learned features of various models, whereas the late fusion method combines the confidence scores of all individual models. Experiments show the superiority of the proposed method over individual methods. Both fusion mechanisms outperform baselines by a large margin. In addition, the early fusion model consistently outperforms the late fusion mechanism on the two benchmark datasets. In particular, the early doctor consultation-inspired model outperforms all individual models by a large margin, i.e., 3.03 and 1.86 in terms of accuracy on the UIT COVID-19 and chest X-ray datasets, respectively.

In the future, we intend to extend our model for different diseases. Moreover, we plan to explore different kinds of medical imaging, such as CT scans or MRI. The proposed method also has the potential to integrate additional individual models to better recognize disease from an input X-ray image. The proposed method addresses the classification problem. Therefore, we intend to investigate the effectiveness of the proposed method on various tasks, such as semantic segmentation or instance segmentation in medical images.

## Figures and Tables

**Table 1:** Comparison of related works.

Method	Year	Classification	Lung Segmentation	Refinement/Remarks
Shibly et al. [10]	2020	VGG-16	No	No
Sethy et al. [12]	2020	ResNet-50, SVM	No	No
Abraham et al. [14]	2020	Xception, Bayes Net	No	No
Mei et al. [16]	2020	ResNet-18, MLP	No	No
Tuncer et al. [18]	2020	Local Binary Pattern, SVM	No	IRF-based feature selection
Hemdan et al. [20]	2020	DenseNet-201	No	No
Ardakani et al. [22]	2020	ResNet-101	No	No
Waheed et al. [25]	2020	CNN	No	GAN-based data augmentation
Tartaglione et al. [29]	2020	ResNet-18	Yes	Segmented lung
Perumal et al. [32]	2021	CNN	No	Transfer learning with Haralick features, CT scan
Teixeira et al. [27]	2021	InceptionV3	Yes	Segmented lung
Balaha et al. [30]	2021	CNN	Yes	Geometric transformation-based data augmentation, segmented lung, genetic algorithm
Baghdadi et al. [31]	2022	CNN	No	Sparrow search algorithm, CT scan
Ours	2022	CNN, SVM	No	Doctor consultation-inspired fusion

**Table 2:** Table of abbreviations/acronyms used in this paper.

Abbreviation	Meaning
CXR	Chest X-ray
CT scan	Computed tomography scan
MRI	Magnetic resonance imaging
RT-PCR	Real-time reverse transcription-polymerase chain reaction
ARDS	Acute respiratory distress syndrome
CNN	Convolutional neural network
ResNet	Residual neural network
HRNet	High-resolution network
DenseNet	Dense convolutional network
SVM	Support vector machine

**Table 3:** Table of symbols used in this paper.

Symbol	Meaning
n	The number of models (doctors)
$p_{i}$	The prediction score of model i
m	The number of classes, such as COVID, pneumonia, and normal
[.||.]	Concatenation operation
$f_{late}$	The classification function for late fusion
$x_{i}$	The deep-learned features extracted from model i
$f_{early}$	The classification function for early fusion
$l_{2}$ norm	The square root of the inner product of a vector with itself

**Table 4:** Ablation study on the UIT COVID-19 dataset [39]. The performance of individual doctor models and two implementations of doctor consultation-inspired models. The top-two methods are marked in **red** and **blue**, respectively.

Model	Accuracy	Precision	Recall	*F*1 Score
ResNet-18	89.39	89.70	89.39	89.34
ResNet-50	90.15	90.18	90.15	90.09
ResNet-101	90.91	90.87	90.91	90.87
ResNet-152	90.53	90.64	90.53	90.46
ResNeXt-101	90.53	90.60	90.53	90.46
DenseNet-169	90.53	92.05	92.05	92.03
DenseNet-201	91.67	91.82	91.67	91.64
HRNet-W48	91.29	91.29	91.29	91.26
**Late Consultation**	**92.42**	**92.42**	**92.42**	**92.42**
**Early Consultation**	**94.70**	**94.70**	**94.70**	**94.70**

**Table 5:** Ablation study on the chest X-ray dataset [40]. The performance of individual doctor models and two implementations of doctor consultation-inspired models. The top-two methods are marked in **red** and **blue**, respectively.

Model	Accuracy	Precision	Recall	*F*1 Score
ResNet-18	89.75	90.42	89.75	89.93
ResNet-50	92.47	92.49	92.47	92.42
ResNet-101	90.53	90.49	90.53	90.37
ResNet-152	89.52	89.45	89.52	89.37
ResNeXt-101	92.55	92.54	92.55	92.49
DenseNet-169	92.00	91.95	92.00	91.95
DenseNet-201	93.17	93.16	93.17	93.12
HRNet-W48	92.62	92.60	90.62	92.56
**Late Consultation**	**93.94**	**93.92**	**93.94**	**93.93**
**Early Consultation**	**95.03**	**95.03**	**95.03**	**95.03**

**Table 6:** Comparison with state-of-the-art baselines. The top-two methods are marked in **red** and **blue**, respectively.

Method	UIT COVID-19 Dataset			Chest X-ray Dataset		
	Accuracy	Precision	Recall	Accuracy	Precision	Recall
Shibly et al. [17]	90.24	90.24	90.24	90.68	90.60	90.68
Sethy et al. [18]	90.15	90.18	90.15	92.47	92.49	92.47
Abraham et al. [38]	88.24	89.28	88.24	92.00	91.99	92.00
Mei et al. [39]	89.39	89.70	89.39	89.75	90.42	89.75
Hemdan et al. [32]	91.67	91.82	91.67	93.17	93.16	93.17
Ardakani et al. [22]	90.91	90.87	90.91	90.53	90.49	90.53
**Late Consultation**	**92.42**	**92.42**	**92.42**	**93.94**	**93.92**	**93.94**
**Early Consultation**	**94.70**	**94.70**	**94.70**	**95.03**	**95.03**	**95.03**

## Data Availability

COVID-19 dataset is available at https://github.com/nguyenvd-uit/uit-together-dataset/blob/main/COVID-19.md (accessed on 10 November 2022). Chest X-ray dataset is available at: https://www.kaggle.com/datasets/prashant268/chest-xray-covid19-pneumonia (accessed on 10 November 2022).
